# Reducing the health disparities of Indigenous Australians: time to change focus

**DOI:** 10.1186/1472-6963-12-151

**Published:** 2012-06-10

**Authors:** Angela Durey, Sandra C Thompson

**Affiliations:** 1 Curtin Health Innovation Research Unit, Curtin University, Perth, 6845, Western Australia; 2 Combined Universities Centre for Rural Health, University of Western Australia, Fitzgerald Street, Geraldton, 6530, Western Australia

## Abstract

**Background:**

Indigenous peoples have worse health than non-Indigenous, are over-represented amongst the poor and disadvantaged, have lower life expectancies, and success in improving disparities is limited. To address this, research usually focuses on disadvantaged and marginalised groups, offering only partial understanding of influences underpinning slow progress. Critical analysis is also required of those with the power to perpetuate or improve health inequities. In this paper, using Australia as a case example, we explore the effects of ‘White’, Anglo-Australian cultural dominance in health service delivery to Indigenous Australians. We address the issue using race as an organising principle, underpinned by relations of power.

**Methods:**

Interviews with non-Indigenous medical practitioners in Western Australia with extensive experience in Indigenous health encouraged reflection and articulation of their insights into factors promoting or impeding quality health care to Indigenous Australians. Interviews were audio-taped and transcribed. An inductive, exploratory analysis identified key themes that were reviewed and interrogated in light of existing literature on health care to Indigenous people, race and disadvantage. The researchers’ past experience, knowledge and understanding of health care and Indigenous health assisted with data interpretation. Informal discussions were also held with colleagues working professionally in Indigenous policy, practice and community settings.

**Results:**

Racism emerged as a key issue, leading us to more deeply interrogate the role ‘Whiteness’ plays in Indigenous health care. While Whiteness can refer to skin colour, it also represents a racialized social structure where Indigenous knowledge, beliefs and values are subjugated to the dominant western biomedical model in policy and practice. Racism towards Indigenous patients in health services was institutional and interpersonal. Internalised racism was manifest when Indigenous patients incorporated racist attitudes and beliefs into their lived experience, lowering expectations and their sense of self-worth.

**Conclusions:**

Current health policies and practices favour standardised care where the voice of those who are marginalised is often absent. Examining the effectiveness of such models in reducing health disparities requires health providers to critically reflect on whether policies and practices promote or compromise Indigenous health and wellbeing - an important step in changing the discourse that places Indigenous people at the centre of the problem.

## Background

Over the decades, colonised countries including Australia, Aotearoa/New Zealand, Canada, the United States and Latin America have produced research evidence and government policies identifying the need to improve the poor state of Indigenous health. However, success in eliminating disparities has been limited. Indigenous peoples have worse health than non-Indigenous populations, are over-represented amongst the poor and disadvantaged, and have lower life expectancies [1-4], disparities which are greater in Australia than in other developed countries. When the International Decade of the World’s Indigenous Peoples closed in 2004, little progress had been made in improving environmental conditions, health, housing and education, all significant determinants of Indigenous health [5].

Health disparities in access to treatment occur in most major illnesses including cancer, cardiovascular disease, kidney disease and oral health in developed countries such as Australia [6], Aotearoa/New Zealand [7] the United States [8], Canada [9] and other colonised countries such as Ecuador [10]. A recent report on cardiovascular health identifies Indigenous Australians as having twice the in-hospital mortality rate and a 40 % lower rate of angiography and percutaneous coronary interventions than other Australians [11]. They are also six times more likely (age adjusted) to discharge themselves from hospital against medical advice [12]. Other areas of health present a similar picture. The stigma associated with mental illness persists across many cultures and causes harm, and like others, many Indigenous people are reluctant to attend and have inadequate access to health care services [13,14]. In the US, ongoing disparities in income and education and the pervasiveness of poverty all impact negatively on the mental health and wellbeing of American Indian and Alaska Native peoples and lead to early morbidity and mortality [14]. Indigenous populations in Canada, the United States, Australia and Aotearoa/New Zealand are over-represented in prisons [15,16] and those with mental illness constitute some of the most vulnerable groups in society, experiencing further isolation and stigmatisation as a result of their illness and often facing additional risks from racial discrimination [17].

Research on health disparities in developed countries that focuses on disadvantaged or marginalised groups offers only a partial understanding of influences underpinning slow progress in eliminating health differentials [18]. While the literature often rightly foregrounds the need for Indigenous groups to be ‘empowered’ and ‘resilient’ in the face of racial discrimination, critical analysis is also required of those with power and privilege and the way in which social inequalities are perpetuated through their influence on policies and practices that effectively preserve and reproduce their privilege [18]. When the powerful rather than the disadvantaged are the subjects of study, the focus of the research changes as health care decision-making and implementation come under scrutiny to identify whether the health system is working to improve or undermine the health of Indigenous people [18,19].

In this paper, we explore health care providers’ accountability in improving Indigenous health. We use Australia as a case example to interrogate ‘White’, Anglo-Australian cultural dominance to show how those in positions of power and privilege - policy makers and health care providers embracing a western biomedical model of health care – can compromise, however unwittingly, the health of Aboriginal and Torres Strait Islander (Indigenous) Australians. We suggest that while Whiteness can refer to skin colour, it also refers to a racialised social structure where health care providers ‘willingly and unwillingly, knowingly and unknowingly’ subjugate Indigenous knowledge (epistemology), beliefs (ontology) and values (axiology) to the hegemonic western biomedical model at the level of policy and practice [20]: 341].

We address the issue in a health care setting in Western Australia using race as a structuring or organising principle in social relations that is underpinned by relations of power [21]. Singleton and Linton [22] suggest that race relates to physical characteristics including skin colour while ethnicity refers more to cultural factors such as language, beliefs and practices of a particular population group. They argue that meanings attributed to race are socially constructed often impacting on ethnicity where ethnic markers such as music and food become racialised in the dominant, White culture. Reference is made in the US to ‘Black music’ or ‘Asian food’ with no reference made to ‘White music’ or ‘White food’, implying the normativity and invisibility of ‘Whiteness’.

We acknowledge other important structuring principles such as class and gender, but are persuaded by Singleton and Linton’s [22] argument that race can supersede both in informing how resources and opportunities are allocated. In Australia, Whiteness is the ‘omnipresent norm’, the standard against which differences, or deviations from that norm, are measured, valued and often demeaned [21]: xix]. While Australia has prided itself on its multi-culturalism and being “the land of the fair go”, White, English-speaking Australians are privileged as a group. This position of advantage is linked to the colonisation and dispossession of Indigenous Australians by the British in 1788 when Indigenous rights and occupancy were ignored (“terra nullius”) and sovereignty and “ownership” claimed by the British monarchy whose authority prevailed in institutions, policies and practices [21]. Being White confers structural advantage, usually invisible to those who are White, and operates through a set of cultural practices that shape both the lives of the privileged and the marginalised [23]. While improvements in health and education have occurred for Indigenous Australians over the last 40 years, inequities persist with Whiteness still acting as a powerful social and cultural force that reproduces inequalities [21,24]. This article explores issues related to discrimination based on race towards Indigenous patients at systemic/institutional, interpersonal and internalised levels.

### Racism

We borrow Katz’s [cited in [22]: 42] definition of racism as ‘prejudice + power’. In the Australian context, White Anglo-Australians have the institutional power to convert prejudiced attitudes into policies and practices see[24]. Racism disproportionately affects Indigenous Australians who have collective experiences and memory of abuse, dispossession, insensitivity and are more likely to be discriminated against in housing, education, employment [25], the justice system [26] and health where they are less likely to be offered the range of options for treatment and appropriate care compared to those who are non-Indigenous [27,28]. Current policies and practices are products of colonial processes that have shaped White people’s sense of the way things are and should be, and which incorporate their own entitlement, superiority and established systems within which they can manage their affairs and use resources effectively for their advantage, thus being considered “successful”. Yet Whiteness often remains ‘invisible, natural, normal and unmarked’ [21]: 183] whereas racism towards Indigenous people persists, often unchallenged and unreported in health care contexts [29] despite its damaging effects on health [30,31].

People often view racism solely as referring to interpersonal relations, where a person is treated unfairly in interactions because of race [32]. However, racism that exists systemically and institutionally, where the production, control and access to resources operates to advantage selected racial/cultural groups and disadvantage others, is more insidious [33]. Racism can also be internalised where members of a stigmatised racial group accept attitudes beliefs or ideologies about the inferiority of their own ethnic/racial group, [33], lowering their expectations, performance and sense of self-worth. Paradies [33]: 3] defines this in terms of ‘racialisation’ and suggests that racism as a form of oppression exists in dialectic relationship with anti-racism. We argue that those who are White and privileged also internalise their racial positioning in the social order where they accept as the norm a sense of their own entitlement to goods and services, not easily accessed by those from other racial groups.

### Anti-racism

Anti-racism is conceived as a way to reduce power inequities between privileged and oppressed groups [33] and requires a critical analysis of those in power. The cultural capital inherent in Whiteness, and the fact that it is normalised and unchallenged, largely ensures its lack of interrogation, with no imperative to acknowledge and take responsibility for any complicity in perpetuating inequities or changing beliefs and practices [21,22]. A market driven economy also ensures that we allow and richly reward those who engage in private practice in medical and other health service delivery. This means there are a range of treatment choices for the well-to-do and considerable resources committed to the treatment and care of an increasing aged population (rarely Indigenous Australians), even though many of those treated will form part of the elderly population living with a poor quality of life. Contrast that to the Australia that so often fails to provide a basic level of primary health care to that group who are acknowledged as having the most complex social and health needs with a substantially reduced life expectancy. The gap between Indigenous and non-Indigenous Australians is estimated currently at 11.5 years for males and 9.7 years for females [1] although these figures themselves are considered by many to be low, and subject to bias [34,35]. These circumstances demand not just moral outrage at the state of Indigenous health, but that this is linked to changes in mainstream policies and practices which effectively contribute to reducing health disparities [36].

### Models of care

While many health care providers want to improve service delivery to Indigenous patients, the reality in most health settings is that demands for quality and organisational efficiency compete, so there is often little time or resources allocated to understanding the special needs of Indigenous people and the challenges of providing quality primary and follow-up care. However, this justification of inadequate services needs questioning if real improvements to Indigenous health outcomes are to be achieved. In 2010, the Oxfam Shadow Report on the Australian Government’s progress in improving Indigenous health identified the need for ‘developing a comprehensive, long-term plan of action, that is targeted to need, evidence-based and capable of addressing the existing inequalities in health services’[37]: 4]. For this to be effective, barriers to translating good policies to ‘a plan of action’, including ‘grappl[ing] with the challenges of implementation’ [38]: 31] must be identified and removed to reach that goal.

Aboriginal Community Controlled Health Services (ACCHS) were established in the early 1970s as a response to the poor service Indigenous Australians experienced within mainstream health services, service failure undoubtedly underpinned by racism. ACCHS are run by an Indigenous Board elected from the local community to provide health services for Indigenous Australians within a holistic health model that promotes health, treats illness, fosters community development and support services and provides educational resources for health professionals [39]. However, offering separate primary care services does not redress discriminatory practices occurring in mainstream health services which dominate the health care system in terms of funding, coverage and complex treatment. The responsibility of resolving Indigenous health inequities rests not just with Indigenous people or governments, but also requires a sea-change within the mainstream healthcare system. Health care providers must challenge conventional paradigms which have failed Indigenous people and interrupt processes that blame and externalise problems onto the marginalised ‘other’ [40].

### Accountability for translating health care policies to practice

Several studies state the need for further research to address the problem [41,42]. Yet do we act on the evidence that is available see [30,31,43]? - and how much evidence do we need before we make sustained improvements to health outcomes? Indeed, can collecting more evidence delay tackling the problem? To what extent are Indigenous researchers, policy makers and practitioners included in the process of consultation, designing, planning, implementing and evaluating programs to improve Indigenous health? Is their voice heard in critiques of the western health care system, a stronghold of Whiteness, by identifying inequities in policies and practice and holding the system more accountable to improve health disparities? While there have been calls for more intervention research to directly address the health problems of Indigenous Australians [44], most intervention research falls short, focusing mainly on changing risk factors while failing to address the underlying social and structural determinants that underpin poorer health.

Reducing health inequalities is about fairness and social justice [45]. With Australia currently experiencing an economic boom and mining companies recording profits in the billions of dollars, it is an indictment that Indigenous Australians remain ‘overwhelmingly and unremittingly poor’ [25]: 29], with health significantly worse than other Australians. In the current neoliberal climate, the market has become the general measure of social activities that values and underpins policies of caring for others [46]. This reinforces a belief there is no alternative to neoliberalism. Instead, what is effectively ‘wilful blindness’ underpins the failure to recognise and redress the reality of a balance sheet that shows disproportionate losses for Indigenous people with the benefits accruing to privileged groups, not least already wealthy mining magnates. Losses of traditional land and language, social dislocation and cultural fragmentation all negatively impact on Indigenous culture and health. Evidence from Canada shows that connection to culture is a protective factor in the health of First Nation peoples [47] and similar findings of the protective effect of culture and attachment to country has been found for Indigenous Australians [48,49].

Such evidence highlights the challenges facing decision makers to reduce inequities, improve health and respect Indigenous cultural beliefs and practices in the process. The aim of our study was to interview non-Indigenous medical practitioners experienced at working in the Indigenous health area to identify institutional and interpersonal practices in mainstream health settings that compromised the health of Indigenous Australians, and highlight specific areas for improvement.

## Methods

The authors have worked for many years in a variety of health settings including education and research and have experience in domains such as medicine, public health, policy, nursing and anthropology. Our qualitative methods were informed by the analysis of Wolcott who identified the importance of the researcher’s experience and understanding in interpreting the data [50]. We also drew on the work of de Laine [51] to examine health care practice. Her critical approach to analysis identified power relations and how ‘patterns of domination of individuals and groups … stem from fundamental structures and ideologies of social systems’ [51]:125], that are often accepted as part of the ‘normal’ social order [51]:127]. Our study aimed to supplement research examining barriers to Indigenous Australians’ access and uptake of health care [52,53] and was approved by the Western Australian Aboriginal Health Information and Ethics Committee.

### Data collection

We were interested less in the number of participants recruited and more in achieving high quality insights of experienced doctors, committed to Indigenous health, who could deepen our understanding of the issue. Interviews were considered an appropriate method to gather data and encourage participants to reflect on factors promoting and impeding the delivery of quality health care to Indigenous Australians. Our participants were working in Perth, the state capital of Western Australia, and were selected based on their long experience in service delivery in the Indigenous health sector and because their insights and reflective thinking located the problems of medical care for Indigenous clients within a health system which failed to adequately acknowledge and respond to their patients’ needs.

As Wolcott [50] suggests, qualitative research combines the perspective of both the researcher and participants where researchers bring their own experience, cultural background, values and understandings to help inform the research experience. Recruitment and interviews were conducted by ST, a public heath physician who had worked in Indigenous health for many years. Her experience and observations in this field helped, not only to establish rapport with participants, but also to inform the interview questions and research process because of her familiarity with the context.

Interviews conducted with White, Anglo-Australian medical doctors, two female and one male, were arranged at a time and place convenient to participants and interviewer and occurred over several months until there was repetition of themes. Voluntary consent was provided by participants. Interviews lasted from 40 to 120 minutes and occurred over many occasions with questions relating to the challenges participants experienced providing care for their Indigenous patients and the nature of the difficulties. Participants were encouraged to draw on their experiences and observations to reflect on whether mainstream health services undermine or promote quality care for Indigenous Australians. To add rigour to the research process and identify whether participants’ experiences and observations in interviews resonated with a wider group of practitioners experienced working in Indigenous settings, informal discussions were also held with other Indigenous and non-Indigenous medical colleagues working in Indigenous health research, education and practice on their observations while working professionally in policy, practice and community settings.

### Data analysis

Interviews were transcribed and imported into QSR NVIVO qualitative software package where an inductive, exploratory analysis was undertaken to identify and code key themes which were subsequently revised, modified, developed and refined [50]. Both researchers analysed the interviews independently, discussed and corroborated their findings. Key themes from informal discussions with other Indigenous and non-Indigenous medical colleagues working in the Indigenous health area were identified independently by each researcher, discussed and reviewed to reach consensus. Member checking involved the researchers following up on interviews, and informal discussions offered a means to validate whether participants’ experiences and observations in interviews resonated with those of a wider group of practitioners experienced working in Indigenous health care.

Our observations were influenced not just by the recorded words, but by our own beliefs and values as White health professionals. While our past experience, knowledge and understanding of health care and Indigenous health assisted with interpreting the data, findings from interviews, informal discussions and observations also offered different perspectives so new meanings could emerge and lead to a deeper understanding of the issues. Quotes were selected to illustrate key themes identified in interviews. To ensure confidentiality, no information identifying participants is used in the paper.

Findings were reviewed, interrogated, refined and compared to existing literature on health care to Indigenous people including peer reviewed papers, government documents and grey literature such as unpublished reports. Literature was identified by systematic searching as well as citation snowballing and then examined for the role of broader structural issues such as institutional policies and practices on the health care of Indigenous patients. This approach gave us the opportunity to critically analyse power relations underpinning the systemic, institutional and interpersonal challenges health providers face when caring for Indigenous patients in mainstream settings.

## Results

Our findings deepened our thinking around racism which was a key theme identified by participants that stalled progress in improving Indigenous health. Racism manifested in hospital policies, practices and interactions between hospital staff and Indigenous patients which, over time, led to Indigenous patients perceiving racism in interactions even if it was absent. We have conceptualised racism respectively as institutionalised, interpersonal and internalised. However, we acknowledge these are not discrete categories as they interrelate closely in ways that maintain the status quo.

### Institutional racism

While racism at institutional (or interpersonal) levels may not be intentional [54] its effects are no less debilitating. One example was recording demographic information in hospital. Standard forms are used that do not account for complex Indigenous Australian kinship structures or patients’ mobility between family members at different locations; no space is available to record more than one address or next of kin, particularly relevant for a patient coming to the city from a rural area:

"“There are two fields [on the admission form]: a residential address and a postal address … [The patient’s] current residential address was the [aged care facility]. His postal address would probably be Broome and his real residential address would be One Arm Point … But you can’t put all that information on the system. So what was on the system was a residential address from two years ago which was his sister’s in Perth.”"

There is also confusion about terminology regarding primary care providers which can result in no follow up care due to systemic failure to be culturally sensitive (and accurate) when recording contact details:

"“Aboriginal patients will be asked who their GP is – and some may not know what a GP is - up north in the country they are called doctors. If the clerk deems they do not have a doctor, it is written down as ‘nil GP’ and there is no obligation to challenge that.”"

Discharge letters may then be sent from the tertiary hospital to the wrong primary care provider - or not sent at all, based on inaccurate information recorded on patient admission forms.

"“You would think down the line that would be a clinical issue at discharge but it's not because no-one knows about it… If the address is wrong [the GP] won’t get the letter.”"

This suggests a lack of institutional responsibility around reviewing practices which clearly compromise Indigenous health outcomes. However, when Indigenous patients fail to attend follow-up appointments due to communication breakdown between the hospital and the primary care provider, it is not the health care system that is held accountable for poor quality care and communication:

"“When the patient doesn’t attend you are going to blame the patient and it’s not the patient’s fault. And all the time Aboriginal people get a bad name because they did not attend follow-up appointments.”"

### Interpersonal racism

Interpersonal racism between a health care provider and an Indigenous patient was often subtle yet pervasive. There was a lack of awareness and interest in Indigenous Australians’ lived experience which undermined good communication, relationships and quality health care:

"“[Staff] said they could see they had difficulties communicating with Aboriginal people but were trying hard. But they never think about getting a map to find out where this person comes from, what the local language from that area is. Separation from country [for Indigenous Australians] is a really big deal particularly at the end of life … [Staff need to be] asking the question ‘Who could we get to help talk with you in your language’?”"

This lack of engagement with Indigenous patients was also reflected in the dearth of interpreters for Indigenous languages - none were provided in tertiary hospitals to help patients understand health practitioners’ questions or instructions. Despite interpreting services being available for patients of other different cultural and linguistic backgrounds, including for some European dialects, suggestions for interpreters in Indigenous Australian languages Such exclusion, abrogation of responsibility for poor communication and disregard of Indigenous cultural meanings associated with health practice is disrespectful and constitutes a form of institutional and interpersonal racism, which, however covert, remains uninterrogated despite its negative consequences for Indigenous people [30].

"“had all been knocked back every time [the issue] was raised”."

"“Basically I think it is an issue of mistrust. Australians in an institutional setting (like a hospital) somehow have more confidence in interpreters from [Europe] than they do from an Aboriginal community … There is a sense of things being chaotic, disorganised, how would you know if you have the right answers, whereas there is a sense that other Europeans think like us and would probably know what it was all about. A vague unease.”"

### Internalised racism

We acknowledge the challenges of White researchers exploring internalised racism in Indigenous Australians who have a long history, experience and collective memory of being on the receiving end of mainstream White policies and practices that are racist. The subtle but powerful damaging effects of racism are highlighted when, either individually or collectively, Indigenous Australians accept and internalise beliefs and attitudes about their inferiority and expect to be discriminated against in a range of social contexts including the health setting. However, such knowledge can also provide an opportunity for learning and alert health care providers to be more vigilant about the consequences of their attitudes and practices, even where racism is not intended:

"“One thing I would say about racism in hospitals is that sometimes things seem racist from the patient perspective even when they are not. And I suspect the feeling of racism is as bad as the real thing - in terms of flow on effects. Recently, a patient of mine walked out of an oncology clinic because “other people were being taken in ahead of her”. As a result, she was refusing to continue her medication, refusing all further follow up and was in a really bad way. When I followed this up with the clinic, they said that my patient had arrived 30 minutes early and the patients seen before her had earlier bookings. My patient’s interpretation of this “innocent” situation was based on her life-long experience of manifest indisputable racism. And she was especially vulnerable because of the nature and severity of her illness**.** Sadly, the outcome could have been very bad as she had been responding well to treatment.”"

This scenario suggests a need for sensitivity to how internalised racism can manifest in seemingly innocuous health care interactions. Institutional and interpersonal commitment to respond respectfully in such situations is important to promote Indigenous health and wellbeing so patients feel ‘culturally safe’.

Participants’ observations and experiences illustrated in their quotes raise important questions about how best to address the various layers of racism which clearly intersect. A review of Indigenous patient care is warranted to critically reflect on the effects of policies and practices on Indigenous health and wellbeing.

"“Obviously, hospital staff need to address their racist behaviours but they also need to be much more mindful of how their behaviours can be misinterpreted. In the above situation, someone should have anticipated the potential for misinterpretation and clearly explained [to the patient] that her appointment was third in line…or something similar.”"

## Discussion

Racism towards Indigenous patients was seen by participants as multi-layered: institutional, where bureaucratic requirements did not account for cultural differences; interpersonal, where interactions between health provider and patient discounted Indigenous beliefs and practices; and internalised, where, not only were Indigenous Australians unable to assert their right to adequate information and equitable treatment (the result of a legacy of subjugation, dispossession and disadvantage based on race), but also health providers normalised policies and practices that discriminated against Indigenous Australians. Instead, racism remained invisible and uninterrogated by health providers who were often unaware how it became internalised with resulting negative effects on health outcomes.

Potential limitations of this project included interviews being restricted to three non-Indigenous doctors. However the study’s intention was to focus on the high-quality insights of doctors experienced working in this field rather than a pre-specified, greater number of participants. We were unable to recruit an Indigenous doctor to participate in formal data collection which would have added an important perspective to the paper. Other limitations included focusing on doctors working in the metropolitan area rather than those practicing in rural and remote settings, although all had worked in different Indigenous and/or international settings.

While governments may excel at rhetoric, glossy policy documents and promises, their commitments to reducing discrimination against Indigenous people often lack focus and follow through to ensure sustained improvements to health care practice. Overall, examples abound of inadequate resourcing of Indigenous health commensurate to need: evidence of research not being translated to policy and practice; inefficient and wasteful funding models; competitive rather than cooperative approaches; inequitable treatment regimes between Indigenous and non-Indigenous patients; culturally inappropriate care; inadequate or no cross-cultural education of health care providers; ineffective mechanisms to implement Indigenous health frameworks, poor communication, market-driven health provision; and models of care that focus on body systems and prioritise diagnosis, treatment and cure rather than prevention. Furthermore, short term approaches focusing on education and health promotion targeting “lifestyle diseases” are an ineffective substitute for whole of government commitment to addressing the social determinants of health [30,55,56]. Failure to effectively address Indigenous health problems and interrogate policies and practices that are discriminatory has long-term consequences for Indigenous Australians including in the area of health service delivery.

Mainstream structures and practices to improve health in the advantaged are applied to deeply entrenched non-mainstream problems that further disadvantage Indigenous Australians [57]. Our findings support the view that a western biomedical model of practice delivered to Indigenous patients, particularly from remote areas of Australia where English is not their first language, often leads to cross-cultural misunderstandings regarding disease causation and treatment that end up compromising health.

### Ways forward

While some government policies have attempted to meet the issue by providing frameworks to improve performance and accountability in health services to Indigenous Australians and address ‘health service provider attitudes and practice, communication issues, mistrust of the system, poor cultural understanding and racism’ [58]: 6], progress is slow. We became aware of the frustration committed practitioners may feel in their efforts to work in Indigenous health, often defeated by a system that seems blind and indifferent to the needs of Indigenous patients given their social and cultural circumstances. Externalising the problem of cultural and racial difference onto Indigenous Australians avoids addressing quality health care from institutional and interpersonal perspectives. More specifically, blame for ‘non-compliance’, for example, with medication regimens, is often placed squarely on the Indigenous patient rather than the communication skills of health care providers in explaining instructions. ‘Victim blaming’ by health care systems and providers, however unintended, deflects accountability for the negative effects of ‘one-size-fits-all’ practices that exclude or minimise different cultural understandings and meanings associated with health that deviate from those of the established norms of western epistemology, ontology and axiology. Reducing future occurrences is more likely if these situations are used as a catalyst to critically reflect on organisational and interpersonal practices that disadvantage Indigenous patients and are known to underpin their reluctance to access services [59]. Recognising that the problem of health inequities starts with the privileged and powerful [60] and acting on this understanding rather than blaming Indigenous people for their health problems, poor housing, poor school attendance, or poor retention in employment [56,61] makes strides in the right direction.

### Culturally safe care

A key element to improving Indigenous health care is to provide health services that are ethical, respectful and experienced as culturally safe. The concept and principles of ‘cultural safety’ were developed by Irihapeti Ramsden in Aotearoa/New Zealand to respect Maori culture and highlight the negative effects on health outcomes of colonisation and inequitable power relations in mainstream health services that discriminated against Maori [62]. The current focus in Australia on evidence based diagnosis and treatment of symptoms inhibits a deeper, more complex, nuanced understanding of Indigenous health. For example, how well, in a mental health context, does an empirically based, homogeneous approach work for assessing Indigenous patients, when cross-cultural understandings of mental illness are measured through a western-based prism? The heavy reliance on western psychiatric constructs risks inappropriately privileging western understandings of mental disorders that are familiar to western practitioners but lack meaning to those from culturally and linguistically diverse backgrounds where local understandings are more relevant [63]. A more holistic approach locates disease aetiology in a broader socio-economic and historic context and incorporates culturally and linguistically appropriate health promotion, education, prevention of illness as well as clear explanations of treatment.

Kleinman and Eisenberg’s [64]: 252] seminal work identified how illness is ‘culturally shaped’ where culture impacts significantly not just on illness but also on treatment. They underscore the importance of health practitioners considering the influence of a patient’s cultural beliefs and practices on their construction of illness, integral to providing quality health care and working in partnership with Indigenous people. Non-Indigenous health care providers as key agents of change, go beyond health policies to pragmatically improve systems within mainstream settings and reform ingrained discriminatory practices [65]. This requires supporting health policies and practices which respect cultural difference in mainstream settings and being accountable for changing long-standing discriminatory behaviour to create hospital environments that are safe and enabling for Indigenous people [65,66].

One approach is to focus on culturally appropriate assessment and diagnosis where screening, intervention and monitoring tools for Indigenous populations are developed and used in health care [67-70]. In the area of mental health, Hart et al. [71] challenged dominant approaches to service delivery that discount Indigenous knowledge and blame Indigenous people for their limited understanding of mental illness. Instead, they actively involved Indigenous Australians in developing a culturally validated mental health first aid tool to improve mental health literacy and included information about symptom recognition in a cultural context. This point reiterates the notion that understandings of mental health are culture bound yet not always accounted for in mainstream mental health service delivery [72,73].

### Cultural education

In the last few years, cultural education and training programs to work better with Indigenous clients have burgeoned across the health and education sectors. However, while many educational frameworks are available to increase knowledge, skills and understanding of culturally appropriate care, whether culturally safe, culturally competent or culturally secure, these concepts need critical review as empirical evidence on their long-term effectiveness in changing practice or improving Indigenous health outcomes is limited [74-76].

In order to ensure culturally safe health service environments, both institutional and personal commitment is required. The challenge is to avoid tokenism where a service provider attends a cultural education workshop/lecture/seminar to ‘tick a box’ attesting that they have met institutional requirements to complete an education session on cultural safety. Attending one session is unlikely to result in culturally safe practice and raises questions about the purpose of cultural education in health care settings, the institutional commitment to improving Indigenous health through meaningful, long-term engagement and how this is demonstrated in practice. If the institutional requirements are for health care providers to practise in culturally safe ways when working with Indigenous patients, what criteria are put in place to inform the assessment of culturally safe care and who deems a service provider is delivering health care in culturally safe ways?

Education in culturally safe care also requires self-reflection – where service providers examine the quality of the care they provide to Indigenous patients by interrogating the social, political, historical and cultural contexts that shape their own identity to increase their awareness of power dynamics at structural and inter-personal levels that disadvantage their Indigenous clients [see [62]. Yet powerful socio-cultural groups are often indifferent or reluctant to acknowledge they are part of the problem or to examine their role in disadvantaging Indigenous clients, illustrating Wisniewski’s concept of ‘the averted gaze’[77]:5]. We suggest this is a form of wilful blindness which leads to inaction, maintains the status quo, produces more talk about health inequalities but little fundamental change – in other words, a health system which remains part of a broader system that undermines the health of disadvantaged while “privileged” health professionals continue to meter out medical and lifestyle advice.

Making visible the White privilege that exists in health service discourse and delivery highlights the power differentials inherent in its invisibility [21] and is a step towards a more reflective, ethical process at the levels of health policy and practice that moves towards more equitable delivery of health care. This process develops a critical consciousness that offers a different perspective on caring for Indigenous clients where health care providers suspend disbelief and subjugate their own worldviews that may have discounted the client’s understanding of health and illness, instead becoming attentive to such perspectives. Service providers who identify their own biases and preconceptions may become less likely to impose their values and beliefs on their clients and more likely to challenge the status quo [78]. Health systems must review cross-cultural care where Indigenous people are constructed as the problem [79] and instead focus on system, provider, patient and carer-related policies and practices for their ongoing effectiveness in improving Indigenous health outcomes.

## Conclusions

Turning the gaze to the privileged requires rethinking the focus of care, critically reflecting on whether policies and practices support or undermine Indigenous health, and engaging Indigenous people in the process. Applying mainstream solutions to a non-mainstream problem has done little to reduce health disparities between Indigenous and other Australians [57] despite the culturally blind “universal” approach to health care (epitomised in the often repeated comment “I treat everyone the same”) which has been found wanting. While considering alternative strategies in mainstream health settings to reflect principles of equity and respect to respond to the Indigenous health issue [57], a step forward is to engage and keep Indigenous stakeholders at the table to co-construct policies and programs that are adequately resourced, where both Indigenous and non-Indigenous people are accountable for translating them to practice and evaluating their short and long-term effectiveness in improving health outcomes. This also requires that health practitioners establish and build long-term partnerships with Indigenous communities, developing their capacity to improve health by offering a more collaborative, sustained framework that recognises and respects Indigenous ontology epistemology and axiology. This approach repositions more equitably the role and power of White people in decolonising institutional practices that discriminate against Indigenous Australians and instead implementing those that improve health outcomes. This devolution of power is appropriate and necessary for solutions to the complex circumstances that created the disparities that exist, and enables Indigenous and non-Indigenous people to come together as ‘individuals, as a society and as a health system to form true and equitable partnerships’ in the healing process [80]: 498].

We have the opportunity to honour Indigenous people’s ‘equal right to the enjoyment of the highest attainable standard of physical and mental health’ [81]: 53] through policies that translate to sustained improvements to practice. What is needed is a commitment to refute tokenism and engage deeply with ethical practice at many levels. It is essential that standardised models of care are examined for their effectiveness in reducing disparities in Indigenous health. Encouraging health care providers to critically reflect on how normative, White privilege can reproduce inequities in Indigenous health care is an important step to changing the discourse that places Indigenous people at the centre of the problem in Australia along with the failed service approaches that exist.

## Competing interests

The authors declare they have no competing interests.

## Authors’ contributions

AD contributed to the conception and design of the study including the data collection, analysis and interpretation of data. She drafted and reworked the manuscript and approved the final version for publication. ST conceived of the study and contributed to its design, data collection, analysis and interpretation. She critically revised the manuscript for important intellectual content and approved the final version for publication. All authors read and approved the final manuscript.

## Pre-publication history

The pre-publication history for this paper can be accessed here:

http://www.biomedcentral.com/1472-6963/12/151/prepub
